# Case report: coeliac disease as a cause of secondary failure of glibenclamide therapy in a patient with permanent neonatal diabetes due to *KCNJ11*/R201C mutation

**DOI:** 10.1007/s00125-021-05454-y

**Published:** 2021-05-14

**Authors:** Dario Iafusco, Angela Zanfardino, Alessia Piscopo, Francesca Casaburo, Angelica De Nigris, Salvatore Alfiero, Giuseppina Russo, Mattia Arenella, Maria Cecilia Russo, Fabrizio Barbetti

**Affiliations:** 1 Department of Pediatrics, University of Campania ‘Luigi Vanvitelli’, Naples, Italy; 2 Department of Experimental Medicine, University of Rome Tor Vergata, Rome, Italy

**Keywords:** Coeliac disease, Glibenclamide therapy, *KCNJ11* mutation, Permanent neonatal diabetes, Secondary failure

*To the Editor*: Activating mutations in the ATP-regulated K^+^(K_ATP_)channel genes *KCNJ11* and *ABCC8* cause neonatal diabetes mellitus (NDM); patients with these mutations may respond to oral sulfonylureas [1, 2]. Recently, we have shown that sulfonylurea therapy in 83 patients with permanent NDM (PNDM) associated with *KCNJ11* mutations maintained its full efficacy in 75 patients (93%) over a period of 10 years, while six needed some additional insulin [3]. To date, while non-responders have been described [4] no severe secondary failure has been reported.

## Methods: clinical case

**Fig. 1:**
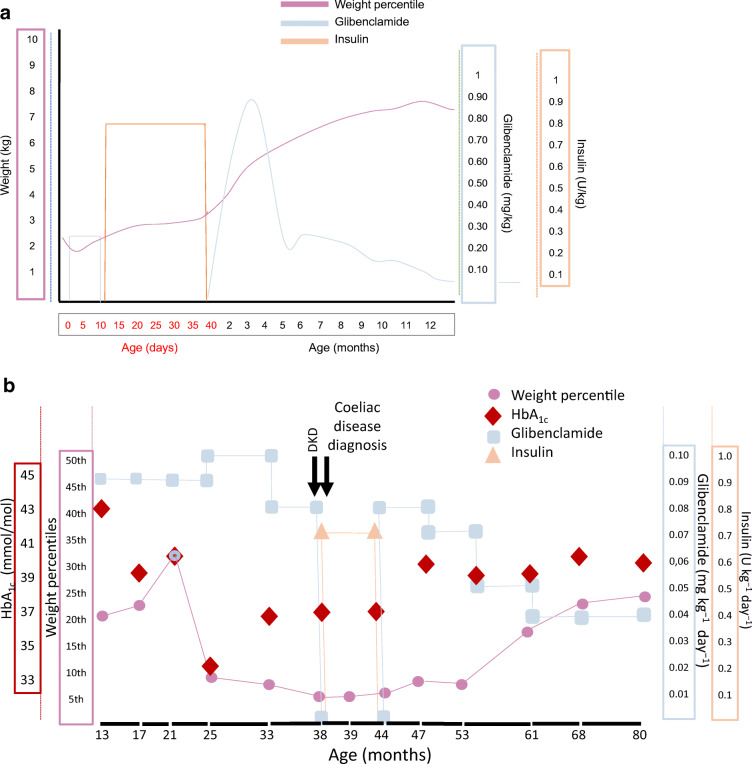
(**a**) Clinical course during the first year of life. (**b**) Clinical course after the first year of life. Effect of glibenclamide in the patient before the diagnosis of coeliac disease, at the time of the DKA episode and after the coeliac disease diagnosis and introduction of gluten-free diet therapy

An infant born small for gestational age (SGA; 2100 g, <3rd centile), the daughter of a PNDM patient carrying the *KCNJ11* mutation R201C, was diagnosed with neonatal diabetes right after birth (blood glucose fluctuating between 4.4 and 22.2 mmol/l (80 and 400 mg/dl). The markers of autoimmune diabetes (autoantibodies to GAD, islet antigen 2 [IA2] and zinc transporter 8 [ZnT8] and insulin autoantibodies [IAA]) were negative. Family history prompted us to commence treatment with the sulfonylurea glibenclamide at a dose of 0.36 mg kg^–1^ day^–1^ (in three administrations) on day 3 of life, before the report confirming that she had inherited the paternal mutation (obtained at 12 days of life). However, because of a failure to thrive (steadily decreasing body weight, down to 1950 g) and unsatisfactory metabolic control, insulin was added to sulfonylureas for about a month, resulting in the patient’s weight gain of approximately 180 g per week. At 42 days of life she was finally weaned from insulin injections at the glibenclamide dose of 0.92 mg kg^–1^ day^–1^. She remained in good metabolic control for 3 years, with HbA_1c_ (DCA-2000 Analyzer, Siemens Healtheeners, Germany) below 42 mmol/mol (6%) at every trimestral visit. At 38 months of age (June 2016), she presented with fever, vomiting and biochemical evidence of diabetic ketoacidosis (DKA): arterial pH 7.1, blood glucose 22.2 mmol/l (400 mg/dl), ketonaemia 6 mmol/l, in the presence of a relative decrease of body weight that occurred a few months before the onset of hyperglycaemia. HbA_1c_ 6 months before and at the time of the DKA episode was normal (37 mmol/mol [5.5%]) (Fig. 1). Glibenclamide (dose at the time of hospitalisation: 0.08 mg kg^–1^ day^–1^) was stopped and insulin therapy was commenced according to the Glucose Evaluation Trial for Remission (GETREM) protocol (0.05 U kg^–1^ day^–1^ i.v.) [5]. The parents informed us that there had been no changes in the therapeutic regimen and so we sought the possible causes of metabolic derangement. Informed consent was given and our investigation was carried out in accordance with the Declaration of Helsinki as revised in 2008.

## Results

Blood analysis showed an elevation in aminotransferases (aspartate aminotransferase [AST]: 58 U/l, alanine aminotransferase [ALT]: 46 U/l) and microcytic anaemia (mean corpuscular volume [MCV]: 65 fl, mean corpuscular haemoglobin [MCH]: 19 pg/cell) with hyposideraemia (0.15 μg/ml). An abdomen ultrasound was normal. Further investigations revealed that the patient carried coeliac disease-predisposing HLA haplotypes (HLA DQ2 positive, DQ8 negative) and unequivocally elevated transglutaminase 2 (TG2) IgA (96 U/ml; positive >16 U/ml). Jejunal biopsy confirmed the diagnosis of coeliac disease. A gluten-free diet was prescribed to the patient with normalisation of aminotransferases, blood iron levels and erythrocyte analysis and a substantial decrease of TG2 IgA titre (7.0 U/ml after 1 year; negative <9 U/ml), a sign of adherence to the gluten-free diet [6]. Six months from the start of the gluten-free diet, glibenclamide was re-introduced at the same dose (i.e. 0.08 mg kg^–1^ day^–1^) that was administered at the time of hospitalisation (Fig. 1). Over a period of about 3 years glibenclamide was progressively tapered off to the current dose of 0.04 mg kg^–1^ day^–1^. HbA_1c_ remained stable between 39 and 40 mmol/mol [5.8–5.9%], slightly higher than the values observed immediately before and during insulin therapy for DKA (Fig. 1). At the diagnosis of coeliac disease we again investigated autoimmune diabetes markers (GAD, IA2, IAA and ZnT8 autoantibodies) which were negative, demonstrating the nonautoimmune pathogenesis of diabetes. For this reason, the coexistence of the two diseases in our patient has to be considered accidental.

## Discussion

This case demonstrates how coeliac disease may hamper sulfonylurea therapy in patients with PNDM linked to K_ATP_ mutations, and probably in other patients treated with this category of drugs. We previously described an individual diagnosed with neonatal diabetes caused by the *KCNJ11*/R201H mutation, who was successfully switched to glibenclamide as a young adult [7]. The patient missed follow-up visits for 16 months, and asked for medical advice again when she experienced a worsening of her metabolic control (HbA_1c_ 99 mmol/mol [11.2%]) in parallel with a lack of compliance with her gluten-free diet, as evidenced by the raised TG2 IgA titre from 9.3 to 26.7 U/ml [8]. Of note, her TG2 antibody titre decreased (26.7 to 7.6 U/ml), together with her HbA_1c_ (99 mmol/mol to 55 mmol/mol [11.2% to 7.2%]) and effective glibenclamide dose (0.40 to 0.34 mg kg^–1^ day^–1^) after 6 months of strict gluten-free diet. Secondary failure of sulfonylureas seems to be linked to different causes, from unwise selection of patients or inadequate dosage of the drug [9] to lower age and beta cell function at the start of sulfonylurea therapy [10]. Our data support the notion that coeliac disease can be an additional—and rectifiable—cause of secondary failure. It is known that coeliac disease can impair absorption of levothyroxine, paracetamol (acetaminophen) and practolol [11] and a reduced absorption of glibenclamide was likely at work in our case, as suggested by the dynamics of hyposideraemic microcytic anaemia and metabolic control at the diagnosis of coeliac disease and after a gluten-free diet.

Another important aspect of this case is the patient’s failure to thrive during the attempt to switch to glibenclamide. This patient is the only one, among the 30 patients with *KCNJ11*–PNDM in the Italian dataset (F. Barbetti, unpublished results) who received glibenclamide as early as a few days after birth; this early treatment was partially because of the family history of paternal mutation and, in addition, the mild muscle hypotonia found in this infant that prompted us to perform this early attempt at sulfonylurea therapy, considering that the neurological defects observed in PNDM patients may benefit from early treatment with sulfonylureas. At any rate, these data seem to indicate that, in SGA infants with *KCNJ11*–PNDM, sulfonylureas may prove insufficient to guarantee normal increase of body weight in the first month of life and that insulin therapy and delayed sulfonylurea treatment is desirable until proper body weight is achieved.

In conclusion, a sudden failure of sulfonylurea therapy in a patient who has previously responded well to this category of drugs warrants a search for coeliac disease.

## Data Availability

All the data reported in this article are available on request from the authors.

## Abbreviations

DKA: Diabetic ketoacidosis
IAA: Insulin autoantibodies
IA2: Islet antigen 2
K_ATP_: ATP-regulated K^+^ (channel)
NDM: Neonatal diabetes mellitus
PNDM: Permanent NDM
SGA: Small for gestational age
TG2: Transglutaminase 2
ZnT8: Zinc transporter 8
